# On the composition and temporal dynamics of a snake community at the Cerrado-Amazonia ecotone

**DOI:** 10.7717/peerj.20025

**Published:** 2025-09-23

**Authors:** Arthur Sena, Guarino Rinaldi Colli

**Affiliations:** 1 Departamento de Zoologia, Universidade de Brasília, Brasília, Distrito Federal, Brazil; 2 Laboratório de Biodiversidade do Cerrado, Universidade do Estado de Mato Grosso, Nova Xavantina, Mato Grosso, Brazil

**Keywords:** Ecology, Global change, Savannah, Rain Forest, Ecotone, Reptile

## Abstract

Understanding the factors shaping the temporal dynamics of ecological communities is crucial for biodiversity conservation during the Anthropocene. In this study, we describe the species composition of a snake community within the Cerrado-Amazonia transition, the largest forest-savanna transition zone in the world, and a highly threatened ecosystem in Brazil. We also analyzed community structure over time using temporal diversity indices. The community had a balanced mix of forest and savanna species, with the Dipsadidae family exhibiting the highest diversity, including two potentially new taxa. Our research, conducted through extensive fieldwork and literature records, documented 53 species across seven families. This comprehensive approach provided a detailed picture of the snake diversity in this unique transitional zone. The results corroborate previous observations that snake richness is high during wet seasons and show that the community has complex temporal dynamics tied to seasonal shifts. The snake community was active throughout all seasons, with species turnover higher during the wet and dry seasons, marked by a high appearance rate during wetter periods and a high disappearance rate of species during the dry season. The community exhibited positive covariance among species, with each species fluctuating independently over time. However, ongoing deforestation in the region may impact species abundance. These characteristics may be attributed to the unique ecological pressures and adaptations of the transition zone. The presence of species with diverse distribution patterns and habitat preferences underscores the environmental and biogeographic complexity of the region, indicating that the Cerrado-Amazonia transition serves as a critical habitat for a wide range of snake species. We also identified two potential new species, emphasizing the urgent need for further research on the effects of environmental changes on local snake populations and biodiversity. Our findings contribute to understanding Neotropical patterns of herpetofauna diversity and underscore the importance of protecting transitional ecosystems. As these areas face increasing threats from human activities, understanding their ecological dynamics becomes essential for developing effective conservation strategies. Our study serves as a call to action for preserving the unique biodiversity of the Cerrado-Amazonia transition zone and mitigating the adverse impacts of anthropogenic changes.

## Introduction

Temporal community dynamics—shifting species composition and abundance over time—is central to appreciating community stability mechanisms (Hallett et al., 2014). Significant patterns in these dynamics include seasonal variations, successional changes following disturbances or environmental shifts, and long-term changes resulting from climate change and habitat alteration. Such temporal shifts reflect the community’s ability to respond to environmental changes and perturbations; therefore, they are essential for understanding ecological resilience, biodiversity maintenance, and ecosystem function (Paine, Deasey & Duthie, 2018). Moreover, such patterns reveal the species’ adaptive strategies for survival and thriving under varying conditions. Ultimately, selection, speciation, drift, and dispersal determine local communities’ species richness and composition patterns (Vellend, 2010). Selection reflects the deterministic outcome of local species interactions and the environment; speciation, the influence of processes operating at broader scales that affect the regional species pool; drift, the role of stochastic or neutral processes; and dispersal, the flow of individuals and species among local communities (Ricklefs, 1987; Wilson, 1992; Hubbell, 1997). Disentangling the ecological and evolutionary processes that shape community dynamics is crucial for biodiversity management and conservation during the Anthropocene (Gotelli et al., 2017; Magurran et al., 2018).

Alpha (α) and beta (β) diversity play crucial roles in understanding how biodiversity at different scales contributes to community stability across temporal gradients (Hallett et al., 2014; Magurran et al., 2018). α-diversity, measured as species richness within each location, contributes to stability through the portfolio effect, where the presence of more species buffers the community against environmental fluctuations (Tilman, Reich & Knops, 2006). β-diversity, however, captures the variation in species composition between different locations (Magurran, 2004). It highlights how communities change across spatial–temporal gradients, which is crucial for compensatory dynamics where different species respond oppositely to environmental changes, thus stabilizing the community (Fitzpatrick et al., 2013). Together, α- and β-diversity provide a comprehensive view of how local species richness and regional species turnover interact to enhance species community stability under varying environmental conditions (Guerin, Biffin & Lowe, 2013; Alvarenga et al., 2017).

In transitional regions between ecosystems, environmental filters can variably affect α diversity, either increasing or decreasing, depending on the biological group and ecological processes involved in species selection (Van der Maarel, 1990). Environmental filters promote the coexistence of species with similar traits that arise from shared ancestry or convergent evolution, whereas competition works in the opposite direction, limiting the coexistence of similar species (Gao & Reitz, 2017). This outcome stems from closely related species being more similar, leading to phylogenetic clustering through environmental filters, whereas competition often results in phylogenetic overdispersion (Webb et al., 2002). Additionally, community composition over time can be influenced by predator–prey dynamics, as predation is a powerful agent of natural selection. These dynamics are shaped by ecological and evolutionary feedback, species mobility, the predators’ perceptual range for detecting prey, and opportunistic interactions in space and time (Colombo et al., 2019).

Snakes are good models for ecological studies because they are powerful predators in their habitats, and dietary specialization is the primary factor shaping species diversity and community structure (Title et al., 2024). Whereas some species are sedentary, sit-and-wait predators ambushing lizards, rodents, and amphibians (Sazima, 1992; Turci et al., 2009), others are agile and specialized for arboreal habits, with a less robust body, a more elongated tail, and can actively hunt birds, bats, and frogs (Sazima & Marques, 2007). Due to their low abundance, cryptic habits, and diet directly influenced by the resources available in the ecosystem over time, projects involving snake community dynamics are scarce compared to other animal groups, requiring long-term, continuous studies (Vitt & Vangilder, 1983; Steen, 2010).

In South America, several studies concerning snake communities revealed that abundance and richness are related to seasonality, being lower during drier months (Duellman, 1978; Strüssmann & Sazima, 1993; França & Braz, 2013). Regarding spatial–temporal dynamics, snake community structure strongly correlates with environmental factors (De Fraga et al., 2018; Piatti et al., 2019). In the Brazilian Amazonia, snake communities are highly heterogeneous in species composition, with high turnover rates explained by β-diversity and a limited number of species shared between local communities (Frazão et al., 2020). In the Pantanal, snake community composition is influenced more by the flood pulses, whereas forest cover influences β-diversity (Piatti et al., 2019). In the Atlantic Forest, the activity patterns of snakes are positively influenced by daily temperature and relative humidity during the rainy season, likely due to interactions with biotic and abiotic variables, such as prey availability and latitude (Jesus et al., 2023).

Herein, we examine the factors influencing the temporal dynamics of a snake community near the Cerrado-Amazonia transition, with a focus on changes in species composition and seasonal turnover. The Cerrado biome has two well-established seasons: a dry season from May to September and a wet season from October to March (Nimer, 1989). During the rainy season, there is a significant increase in the richness and abundance of anurans (Oda, Bastos & Sá Lima, 2009) and lizards (Godinho, 2019), many of which are preyed upon by snakes and can act as an environmental filter for species occurrence and replacement over time. We hypothesize that biotic mechanisms contribute to community stability, with the relative importance of these mechanisms varying according to seasonal patterns. For instance, negative species covariance can enhance stability by driving trade-offs among species that respond differently to environmental conditions (Ives, Gross & Klug, 1999; Loreau & de Mazancourt, 2013). In this context, compensatory dynamics may be key in sites with highly variable precipitation (Yachi & Loreau, 1999; De Mazancourt et al., 2013). On the other hand, species richness can lead to a “portfolio effect” by distributing a community property across a greater number of species. This distribution reduces the relative fluctuation of the community compared to the fluctuations of individual species, thereby contributing to community stability (Tilman, Lehman Clarence & Bristow Charles, 1998; Lehman & Tilman, 2000; Hallett et al., 2014). Therefore, our objectives are to (1) describe the snake species composition in a locality at the Cerrado-Amazonia ecotone, (2) assess whether patterns of species richness and turnover differ between the rainy and dry seasons, and (3) explore indicators of community stability over time based on temporal patterns in species synchrony and covariance.

## Materials & Methods

### Study area

The Cerrado-Amazonia transition connects the two largest biomes in South America and comprises a complex mosaic of savannas and forests (Ratter et al., 1973; Marques et al., 2019). It provides an ideal setting to investigate the role of α- and β-diversity in shaping the space–time patterns of species communities. Due to the high degree of land conversion for anthropogenic purposes (mainly agriculture, livestock, wood extraction, and fire regimes), the transition is also known as the “Brazilian Deforestation Arc” (Fearnside, 2005; Nogueira et al., 2008; Matricardi et al., 2013). Therefore, the Cerrado-Amazonia transition presents a unique opportunity to understand how ecological communities are structured due to environmental instability resulting from the contact between two or more ecosystems (Van der Maarel, 1990) and anthropogenic disturbances to the landscape.

**Figure 1:**
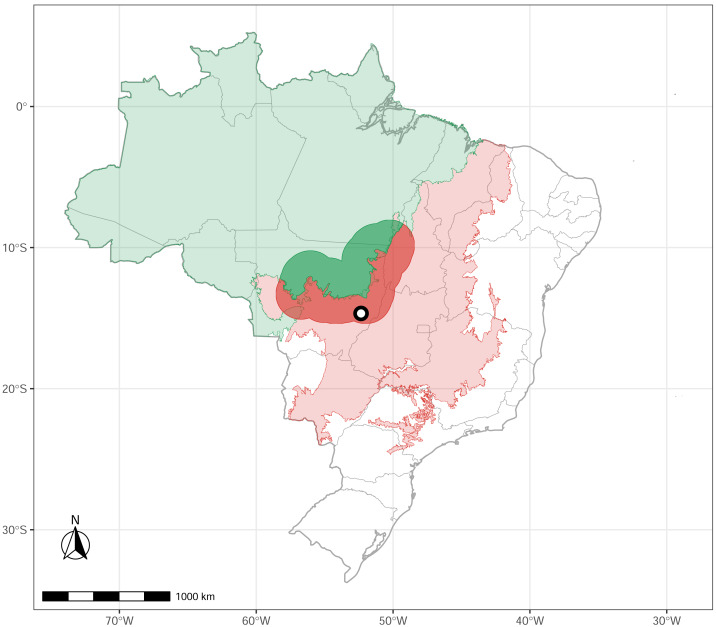
Study site. The green polygon represents the Brazilian Amazonia biome, and the red polygon indicates the Cerrado biome. The circle marks the location of Nova Xavantina, Mato Grosso, situated within the Cerrado–Amazonia ecotone. Transition polygon adapted from Marques et al. (2019).

We conducted a survey in Parque Municipal do Bacaba (14°42′24″S, 52°21′9″W), located in the municipality of Nova Xavantina, Mato Grosso state, in the western portion of the Cerrado biome, near its boundary with Amazonia (Fig. 1). The vegetation within the park comprises “*cerrado* sensu stricto” (open canopy, warmer, dry) with patches of “*cerradão*” (dense woodland savanna, cooler, moister), “*cerrado rupestre*” (saxicolous vegetation), and gallery forest (wet, close canopy, dense woodland forest, water associated). The local climate is markedly seasonal, with a dry season from May to September and a wet season from October to April (Nimer, 1989). The elevation is 340 m above sea level, with annual precipitation ranging from 1,300 mm to 1,500 mm and a mean annual air temperature of 24 °C (Morandi et al., 2020). For a detailed description of the study site, see Alvarenga et al. (2017).

### Sampling

We conducted standardized sampling from May 2018 to October 2020 along a gradient from ‘cerradão’ to ‘cerrado sensu stricto,’ but stopped in October 2020 due to the COVID-19 pandemic. Between November 2020 and December 2022, we documented additional species through occasional field visits, contributions from other researchers, and reports from residents. We used these opportunistic records solely to enhance the final species list and excluded them from the temporal analysis, as they were not obtained through systematic sampling. Our standardized sampling relied on 25 pitfall-trap arrays installed at 30 m intervals along a linear transect of approximately 750 m across a forest-savanna gradient (for details, see Alvarenga et al., 2017). Traps were placed in 2015 as part of a long-term study to monitor the herpetofauna at Parque Municipal do Bacaba. Each pitfall-trap array consisted of three 35-L plastic buckets buried with their openings flush with the ground surface and arranged in a Y-shape. A central bucket was connected to three peripheral buckets by a guide fence made of a galvanized plate, totaling 100 buckets across the sampling area. To assess temporal dynamics, we opened traps for one week each month during all sampling years, totaling 210 sampling days—105 during the wet season and 105 during the dry season. We also actively searched for snakes when traps were opened. We categorized records into (1) active search, (2) pitfall traps, (3) road-killed specimens, and (4) sightings by third parties; the last two included records inside Parque Municipal do Bacaba and in the vicinity of Nova Xavantina. Active searches for snakes were conducted by two researchers across all 210 sampling days, with each researcher surveying for 4 h per day, resulting in a total survey effort of 1,680 person-hours. We also considered encounters by third parties and road-killed specimens outside the week of trap opening. We photographed, identified, and, when necessary (*e.g.*, a low number of specimens or no specimens available from the study site at biological collections), euthanized the snakes with lethal doses of sodium thiopental. We then fixed the animals in 10% formaldehyde and subsequently stored them in 70% alcohol. In these cases, we collected tissues, stored them in cryotubes with 99% alcohol for subsequent molecular analyses, and deposited them in the Coleção Herpetológica da Universidade de Brasília (CHUNB). We returned any surviving specimens to the same spot where they were captured. All activities were conducted under “Licença Permanente para Coleta de Material Zoológico 13324-1” from SISBIO issued to GRC.

We utilized the Atlas of Brazilian Snakes to supplement the species list and document their distributions in the South American ecoregions (Nogueira et al., 2019). Next, we classified species distributions following Valdujo et al. (2012): (1) endemic to Amazonia, *i.e.,* species restricted to Amazonia or occurring in marginal areas of neighboring ecoregions; (2) endemic to the Cerrado, *i.e.,* restricted to the Cerrado (same approach above); (3) widely distributed, *i.e.,* occurring in more than two ecoregions; (4) Amazonia-Cerrado, *i.e.,* widely distributed in Amazonia and the Cerrado; (5) Amazonia-Atlantic Forest, *i.e.,* widely distributed in Amazonia and the Atlantic Forest; (6) Caatinga-Cerrado, *i.e.,* widely distributed in the Cerrado and Caatinga; and (7) Cerrado-Atlantic Forest, *i.e.,* widely distributed in the Cerrado and Atlantic Forest.

### Data analyses

We evaluated the sampling effort and sample completeness for each season using diversity estimates based on a specified number of individuals or sampling units rather than species (Hsieh, Ma & Chao, 2020). The method calculates diversity estimates of different orders q, sample coverage, and associated statistics. The order q determines the sensitivity of the diversity metric to species abundance, with *q* = 0 corresponding to species richness. In our dataset, the extrapolation endpoint was set by default to twice the number of individuals recorded in each sampled season. The function then generates rarefaction and extrapolation curves, illustrating how species richness and diversity estimates increase with sampling effort. This approach enables us to assess sampling completeness and predict the number of additional species that might be expected with increased effort (Chao et al., 2014). We also calculated sample coverage for each season separately to assess the completeness of our inventory, which represents the proportion of the total community that is captured by the sample. High coverage values indicate that most individuals belong to species already detected, suggesting that additional sampling would yield few new species (Chao & Jost, 2012). To produce the site map, we utilized the maptools package (Bivand & Lewin-Koh, 2011; Yu, 2024) and generated graphics using ggplot2 (Wickham, 2016). To access the precipitation data of each season sampled, we downloaded the mean values for each month of the study from the Instituto Nacional de Meteorologia—INMET (https://portal.inmet.gov.br) and then calculated the mean values for each dry and wet season.

We analyzed the capture history of each snake species using the codyn package (Hallett et al., 2016) to assess temporal effects on species turnover and community stability metrics. For our temporal analysis, we represented the capture history of each snake species as a binary variable (0 or 1), indicating whether the species was present or absent across seasons. We pooled captures according to the seasons because monthly data was too sparse. We calculated total turnover as the number of species gained and lost, divided by the number of species observed at both time points. We also assessed changes in species composition by calculating the proportion of species that appear or disappear between seasons. Then, we calculated species synchrony and covariance. Synchrony was determined by comparing the variance of the aggregated community with the variance of its components and by analyzing the average correlation of each species with the overall community.

Species synchrony can be measured using different metrics, such as the one developed by Loreau & de Mazancourt (2008), which compares the variance of aggregated species abundances to the sum of individual species variances and is applicable across communities with varying richness or the approach by Gross et al. (2014), which evaluates the average correlation between each species and the rest of the community, offering richness-independence and suitability for null-model significance testing. Values range from −1, indicating species or processes with compensatory dynamics (*i.e.,* perfect asynchrony), to 1, indicating species that respond similarly to environmental changes (*i.e.,* ideal synchrony), with 0 representing species or processes completely unrelated. To detect compensatory dynamics, we assessed species covariance as the sum of all species plus all pairwise species covariances (Schluter, 1984; Houlahan et al., 2007). We also calculated the rate of community change in species composition using Euclidean distances across the entire time series to assess the direction of compositional shifts within the community (Collins, Micheli & Hartt, 2000). Finally, we calculated the mean rank shift (MRS) to indicate the degree of species reordering between the seasons sampled (Collins et al., 2008; Hallett et al., 2016) and produced a rank abundance distribution (RAD) within rank clock plots to visualize species abundance across the entire time sample. We performed all analyses on the R platform (R Core Team, 2025).

## Results

We recorded 146 snakes, comprising 53 species and seven families (Table 1). Species accumulation curves and sample coverage estimates exceeded 50% in most seasons, except for the dry season of 2019 and the rainy season of 2020—the latter not fully sampled due to interruptions caused by the COVID-19 pandemic (see Figs. S1–S2). The family Dipsadidae contained the highest richness (30 species), followed by Colubridae (11 spp.), Boidae (four spp.), Viperidae (three spp.), Elapidae (two spp.), Leptotyphlopidae (two spp.), and Aniliidae (one sp.). The most abundant species were *Dipsas mikanii* (Schlegel, 1837) and *Pseudoboa nigra* (Duméril, Bibron & Duméril, 1854). Species with only one record were *Apostolepis* sp*., Corallus hortulanus* (Linnaeus, 1758), *Drymarchon corais* (Boie, 1827), *Epictia clinorostris* Arredondo & Zaher, 2010, *Erythrolamprus reginae* (Linnaeus, 1758), *Leptophis ahaetulla* (Linnaeus, 1758), *Micrurus frontalis* (Duméril, Bibron & Duméril, 1854), *Erythrolamprus typhlus* (Linnaeus, 1758) and *Dryophylax hypoconia* (Cope, 1860). We observed a balanced number of Cerrado and Amazonia endemics (Table 2). The less representative distribution categories were those with species shared between Caatinga-Cerrado and Cerrado-Atlantic Forest, and most of the species found occur in more than two biomes (*i.e.,* widely distributed).

**Table 1:** Snake species composition of the community sampled in the ecotonal zone.

**Taxon**	**N**	**Habit**	**Habitat**	**Activity**	**Reference**
**ANILIIDAE**					
* Anilius scytale* (Linnaeus, 1758)	–	C, F	FO	N	This study
**LEPTOTYPHLOPIDAE**					
* Epictia clinorostris* Arredondo & Zaher, 2010	1	F	CE, AN	N	This study^*^
*Trilepida fuliginosa* (Passos, Caramaschi & Pinto, 2006)	–	F	CE	N	^*^
**BOIDAE**					
*Boa constrictor* Linnaeus, 1758	8	SA, T	CE, FO, AN	D, N	This study
*Corallus hortulanus* (Linnaeus, 1758)	1	A	AN, FO	D, N	This study
*Epicrates crassus* Cope, 1862	5	T	CE, AN	D, N	This study^*^
*Eunectes murinus* (Linnaeus, 1758)	–	AQ	FO, CE, Aq	D, N	This study
**COLUBRIDAE**					
* Chironius exoletus* (Linnaeus, 1758)	–	A, SA	FO	D	This study
*Chironius flavolineatus* (Boettger, 1885)	9	A, SA	CE, FO	D	This study^*^
*Drymarchon corais* (Boie, 1827)	1	SA, T	AN, CE, FO	D	This study
*Leptophis ahaetulla* (Linnaeus, 1758)	1	A	AN, FO	D	This study
*Mastigodryas boddaerti* (Sentzen, 1796)	2	SA, T	FO	D	This study
*Oxybelis aeneus* (Wagler, 1824)	–	A, SA	CE	D	^*^
*Oxybelis fulgidus* (Daudin, 1803)	–	A, SA	FO	D	^**^
*Palusophis bifossatus* (Raddi, 1820)	3	T	CE, FO	D	This study^*^
*Spilotes pullatus* (Linnaeus, 1758)	3	A, SA	CE, FO	D	This study
*Spilotes sulphureus* (Wagler, 1824)	–	A, SA	FO	D	^*^
*Tantilla melanocephala* (Linnaeus, 1758)	3	C, F	CE, FO	N	This study
**DIPSADIDAE**					
*Apostolepis sanctaeritae* Ferrarezzi, Barbo & Albuquerque, 2005	4	F	AN, CE	D, N	This study
*Apostolepis* sp. ^***^	1	F	CE	D, N	This study
*Boiruna maculata* (Boulenger, 1896)	–	T	CE	N	^*^
*Dipsas mikanii* (Schlegel, 1837)	13	T	AN, CE, FO	N	This study
*Erythrolamprus almadensis* (Wagler, 1824)	4	T	CE	D	This study
*Erythrolamprus poecilogyrus* (Wied, 1825)	10	T	CE, FO	D, N	This study^*^
*Erythrolamprus reginae* (Linnaeus, 1758)	1	T	FO	D, N	This study^*^
*Erythrolamprus typhlus* (Linnaeus, 1758)	1	T	FO	D, N	This study
*Helicops angulatus* (Linnaeus, 1758)	–	AQ	FO, Aq	N	^*^
*Helicops polylepis* Günther, 1861	–	AQ	FO, Aq	N	^*^
*Hydrodynastes bicinctus* (Hermann, 1804)	2	AQ	FO, Aq	D	This study
*Hydrops triangularis* (Wagler, 1824)	–	AQ	FO, Aq	D, N	This study
*Imantodes cenchoa* (Linnaeus, 1758)	–	A, SA	FO	N	^*^
*Leptodeira annulata* (Linnaeus, 1758)	5	A, SA	CE, FO, AN	D, N	This study
*Lygophis paucidens* Hoge, 1953	2	T	AN, CE	D	This study
*Oxyrhopus guibei* Hoge & Romano, 1977	8	T, SA	CE, FO	N	This study^*^
*Oxyrhopus rhombifer* Duméril, Bibron & Duméril, 1854	5	T	AN, CE, FO	N	This study^*^
*Oxyrhopus trigeminus* Duméril, Bibron & Duméril, 1854	3	T	CE	N	This study^*^
*Phalotris nasutus* (Gomes, 1915)	–	F	FO, CE	N	This study^*^
*Philodryas nattereri* Steindachner, 1870	9	T	AN, CE	D	This study^*^
*Philodryas olfersii* (Lichtenstein, 1823)	5	T, SA	AN, CE, FO	D	This study^*^
*Philodryas psammophidea* Günther, 1872^***^	–	T	CE	D	^*^
*Philodryas viridissima* (Linnaeus, 1758)	–	A, SA	FO	D	^*^
*Phimophis guerini* (Duméril, Bibron & Duméril, 1854)	2	C, T	AN, CE	N	This study
*Pseudoboa nigra* (Duméril, Bibron & Duméril, 1854)	12	T	AN, CE	N	This study^*^
*Psomophis joberti* (Sauvage, 1884)	4	T	AN, CE	D	This study^*^
*Adelphostigma occipitalis* (Jan, 1863)	5	T	AN, CE, FO	D	This study
*Dryophylax hypoconia* (Cope, 1860)	1	T	CE	N	This study
*Xenodon merremii* (Wagler, 1824)	4	T	AN, CE, FO	D	This study^*^
*Xenodon rabdocephalus* (Wied, 1824)	–	T	FO	D	^*^
**VIPERIDAE**					
* Bothrops moojeni* Hoge, 1966	2	T	AN, CE, FO	D, N	This study^*^
*Bothrops neuwiedi* Wagler, 1824	–	T	CE	D, N	This study^*^
*Crotalus durissus* Linnaeus, 1758	5	T	AN, CE, FO	D, N	This study^*^
**ELAPIDAE**					
* Micrurus frontalis* (Duméril, Bibron & Duméril, 1854)	1	C	CE, FO	D, N	This study
*Micrurus surinamensis* (Cuvier, 1816)	–	AQ	FO, Aq	N	^*^

**Notes.**

Taxon N number of individuals found during the temporal analysis sampling

Habit A Arboreal AQ Aquatic C Cryptozoic F Fossorial SA Semi-arboreal T Terricolous

Habitat Aq Aquatic AN Anthropic CE Cerrado FO Forest

Activity D Diurnal N Nocturnal

Reference: *, Nogueira et al. (2019); **, Sena, Corrêa & Quintela, 2022. ***, probably new species.

**Table 2:** Biogeographic distribution pattern of snake species recorded in Nova Xavantina, Mato Grosso. Species were classified according to their known distribution ranges in Brazil and neighboring regions.

**Pattern**	**N**
Widespread	37
Endemic of Cerrado	6
Endemic of Amazonia	4
Amazonia-Cerrado	2
Amazonia-Atlantic Forest	2
Caatinga-Cerrado	1
Cerrado-Atlantic Forest	1

In the site studied, two taxa are considered potential new species. *Philodryas psammophidea* Günther, 1872, currently distributed across Argentina, Bolivia, Brazil, and Paraguay, is divided into three recognized subspecies: (1) *Philodryas psammophidea psammophidea*, (2) *Philodryas p. andensis*, and (3) *Philodryas p. lativittata*. The population found here belongs to the latter and, based on molecular and morphological analyses, has shown consistent differences from the other two subspecies. These results support the hypothesis that it represents a distinct lineage deserving elevation to full species status (Salgar, 2019). The genus *Apostolepis* Cope, 1862 is represented by two species in our study, one of which does not match any known combination of diagnostic characters from the described species (pers. obs., Sena & Colli, 2018). This genus has a complex taxonomic history (Colli et al., 2019; Entiauspe-Neto et al., 2019), primarily due to its underrepresentation in herpetological collections. These are small-to-medium-sized and cryptozoic snakes that are rarely detected by traditional search methods, although pitfall traps with drift fences have proven relatively effective in capturing them.

**Figure 2:**
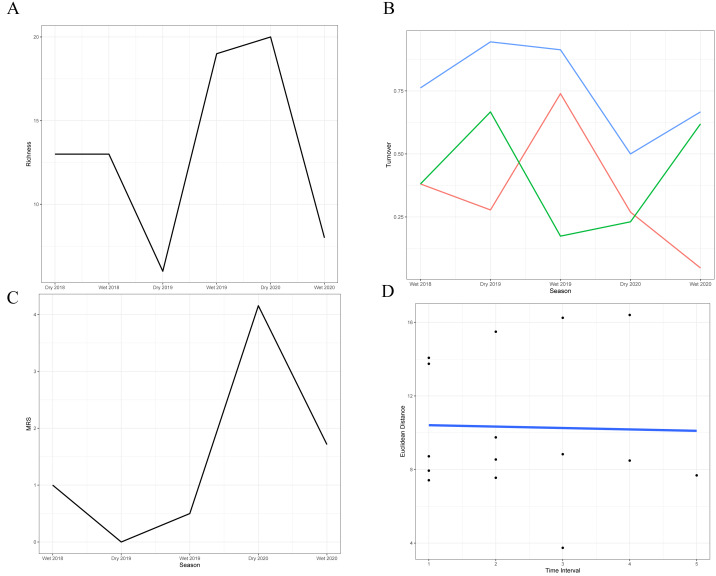
Temporal dynamics of snake community structure across seasons. (A) Variation in species richness across the six sampled seasons (wet and dry seasons from 2018 to 2020). (B) Species turnover between seasons: blue line = total observed turnover, green line = proportion of species disappearing, red line = proportion of species appearing. (C) Reordering of species between consecutive seasons. (D) Temporal trajectory of snake community composition across the 31-month time series. Each point represents the species composition in a given season, based on dissimilarities. The blue line connects seasons chronologically, illustrating the rate (length of steps) and direction (trajectory) of compositional change over time.

The analysis of species turnover across seasons revealed significant variability in the richness of the snake community over time (Figs. 2A–2B; Table 3). The highest turnover rates occurred during the dry season of 2019 (94.4%) and the wet season of 2019–2020 (91.3%), primarily driven by the substantial disappearance and appearance of species, respectively, in comparison with the previous season. These periods indicate significant shifts in community structure, likely in response to the extreme conditions during the dry season and the favorable conditions during the wet season. In contrast, the 2020 dry season showed a lower turnover rate (50%), suggesting relative stability with more balanced species gains and losses. Moderate turnover rates during the wet seasons of 2018–2019 (76.2%) and 2020–2021 (66.6%) reflect periods when the community experienced noticeable but less drastic changes, with the latter characterized by a significant species loss rate that was not fully compensated by new species.

**Table 3:** Seasonal turnover in species composition. Values represent the total turnover in species composition between consecutive seasons, along with the proportion of species that appeared and disappeared.

**Seasons**	**Appearance rate**	**Disappearance rate**	**Total turnover**
Dry 2018–Wet 2018	0.38	0.38	0.76
Wet 2018–Dry 2019	0.27	0.66	0.93
Dry 2019–Wet 2019	0.73	0.17	0.90
Wet 2019–Dry 2020	0.26	0.23	0.49
Dry 2020–Wet 2020	0.04	0.62	0.66

**Figure 3:**
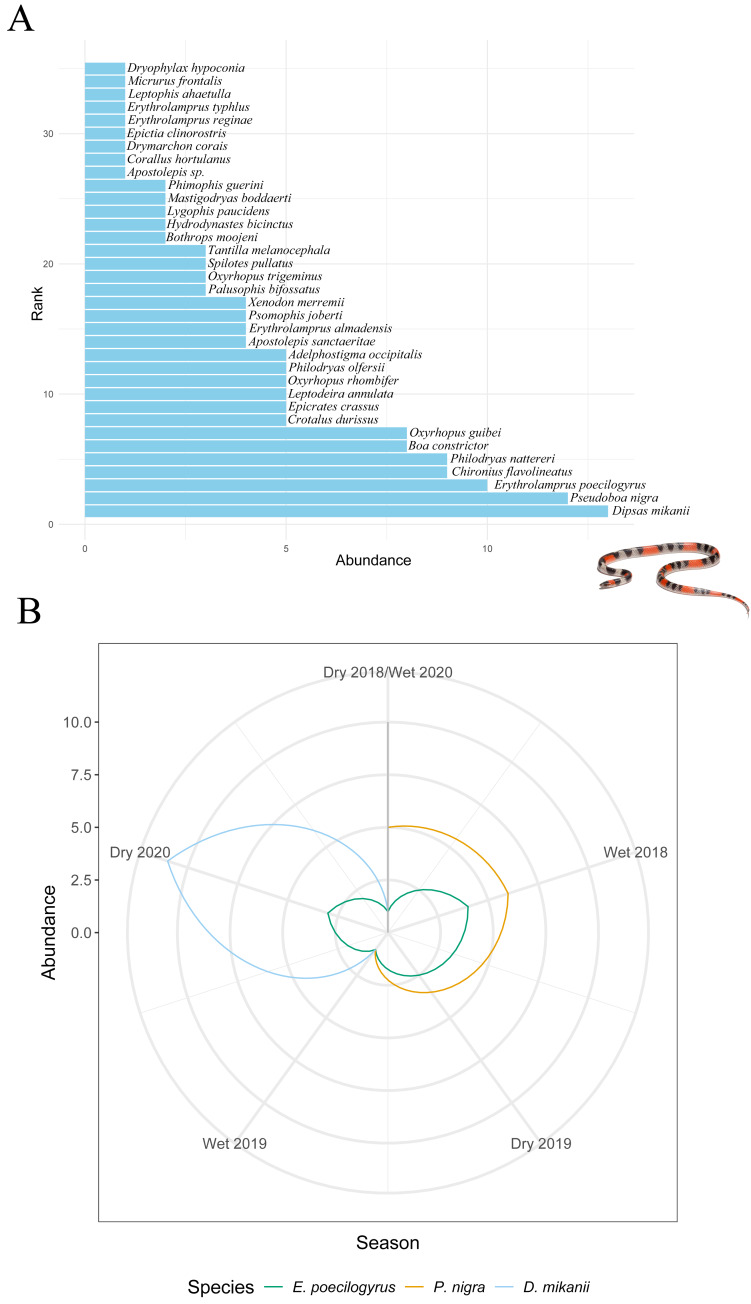
Changes in species abundance and dominance structure. (A) Rank abundance distribution (RAD) for all snake species sampled over 31 months (from April 2018 to October 2020). (B) Rank clock illustrating temporal reordering in the relative abundance of dominant species across seasons. Arrows highlight changes in rank for *Erythrolamprus poecilogyrus*, *Pseudoboa nigra*, and *Dipsas mikanii*. Inset photo: *Oxyrhopus trigeminus* photographed by Arthur Sena.

Stability metrics revealed that the community is stable over time (>1) and exhibited positive covariance among species over time, with no evidence of synchrony (*i.e.,* species fluctuated independently) (Table 4). The mean rank shift indicated that the community converged during the 2019 dry season and began to diverge between this season and the subsequent wet season, reaching its peak divergence in the 2020 dry season (Fig. 2C; Table 5). Additionally, the rate of change in species composition throughout the time series was not significantly different from zero (−0.07) (Fig. 2D), indicating fluctuation or stochastic variation over time. Finally, the RAD plot exhibited the typical pattern for biological communities, where species abundance decreases as species richness increases, highlighting the presence of three dominant species with complex dynamics over the seasons (Fig. 3).

**Table 4:** Community-level metrics over the 31-month time series. Values of temporal synchrony, species covariance, rate of change, and community stability were calculated to assess temporal dynamics of the snake assemblage across all sampled seasons.

**Method**	**Value**
Community stability	1.54
Variance ratio	4.14
Synchrony Loreau	0.19
Synchrony Gross	0.26
Rate of change	−0.07

**Table 5:** Degree of species reordering between consecutive seasons. This table shows the extent of rank changes in species abundances across seasonal transitions, providing a measure of temporal reorganization in species dominance.

**Seasons**	**MRS**
Dry 2018–Wet 2018	1.0
Wet 2018–Dry 2019	0
Dry 2019–Wet 2019	0.5
Wet 2019–Dry 2020	4.15
Dry 2020–Wet 2020	1.71

## Discussion

Our study reveals that, although the ecotonal area sampled is ecologically unstable due to the contact of the two biomes, it is a suitable habitat for widespread snake species and congeneric species from the Cerrado and Amazonia (*e.g.*, *Oxybelis aeneus vs. O. fulgidus*, *Micrurus frontalis vs. M. surinamensis, Philodryas psammophidea vs. P. viridissima*). We also added 23 species to prior lists (Nogueira et al., 2019; Sena, Corrêa & Quintela, 2022), and the combined data were close to the estimated richness of the region. The species richness recorded for Nova Xavantina falls within the expected range for snake communities of the Cerrado (36–70) and Amazonia (56–80) (Cunha & Nascimento, 1993; Bernarde & Abe, 2006; Rios et al., 2017; Colli et al., 2020). Still, six additional species recorded in the vicinities (ca. 150 km) (Nogueira et al., 2019) should probably occur in Nova Xavantina: (1) *Chironius quadricarinatus* (Boiei, 1827) (Cerrado-Atlantic Forest), (2) *Erythrolamprus aesculapii* (Linnaeus, 1758) (widely distributed), (3) *E. taeniogaster* (Jan, 1863) (Amazonia-Atlantic Forest), (4) *Lachesis muta* (Linnaeus, 1766) (Amazonia-Atlantic Forest), (5) *Micrurus lemniscatus* (Linnaeus, 1758) (widely distributed) and (6) *Oxyrhopus petolarius* (Linnaeus, 1758) (widely distributed).

The presence of species endemic to Amazonia and shared between Amazonia and the Cerrado might be linked to the shifting boundaries of these biomes during the climatic fluctuations of the late Pleistocene and Holocene. Throughout this epoch, natural vegetation underwent dynamic cycles of expansion and contraction, with forest intrusions into open areas during warmer and wetter periods and open areas expanding into forested regions during colder and drier periods (Pessenda et al., 2001; Pessenda et al., 2010; Ledo & Colli, 2017). The limited occurrence of only two aquatic species—*Hydrodynastes bicinctus* and *Helicops polylepis*—shared between the Amazonia and Cerrado is likely because of dispersal along watercourses such as the Mortes River, which facilitated movement both by land, *via* forests along riverbanks, and by water (Oliveira-Filho & Ratter, 2002; Ratter, Ribeiro & Bridgewater, 2006). Additionally, the shared species between Amazonia and the Atlantic Forest aligns with postulated ancient connections between these rainforests during the Oligocene and Pleistocene, facilitating the exchange of vertebrate lineages that now inhabit both regions (Ledo & Colli, 2017; Machado et al., 2023). Consequently, relic populations in the ecotone likely reflect the evolutionary history of those species with disjunct distributions between Amazonia and the Atlantic Forest.

**Figure 4:**
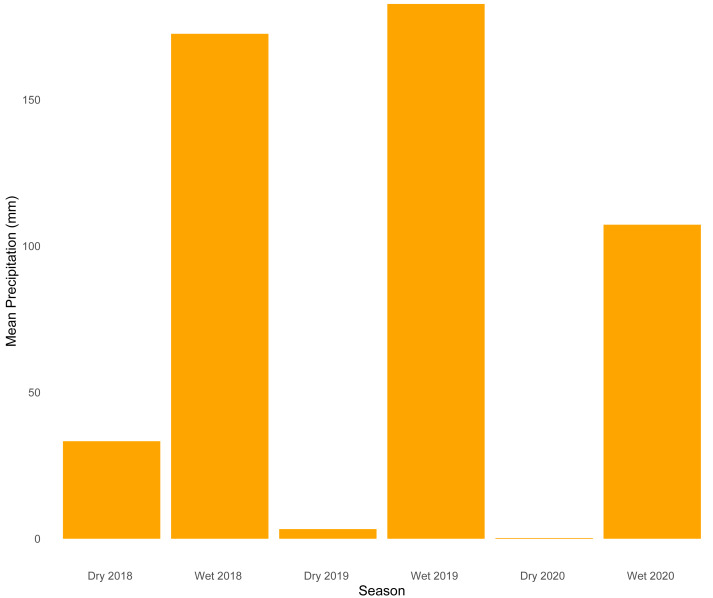
Mean precipitation across sampled seasons. Bar plot showing average precipitation values (mm) for each of the six sampled seasons (wet and dry seasons from 2018 to 2020).

Our analysis of temporal dynamics indicates that this ecotonal community exhibits intricate patterns strongly influenced by seasonal variation. We observed high species turnover during both seasons, with wetter periods characterized by a greater number of species appearing. On the other hand, drier periods were marked by the disappearance of species. The relatively lower sample coverage and species accumulation observed during the dry season of 2019 are likely explained by the combination of ecological and environmental constraints typical of that period. Snakes are among the most challenging vertebrate groups to sample consistently due to their cryptic behaviors, irregular activity patterns, and frequent use of inaccessible habitats—such as subterranean, arboreal, or aquatic environments—which reduce detectability (Turner, 1977; Parker & Plummer, 1987). These challenges are further exacerbated during the dry season, when harsh environmental conditions such as reduced humidity (Fig. 4) could lead to lower prey availability and decreased surface activity, which would likely limit both snake movement and the effectiveness of passive sampling methods. Together, these factors likely contributed to reduced detection probability and explain the lower completeness estimates for that period.

The increased species appearances during the rainy season may reflect broader patterns, as snake diversification over geological time has been associated with dietary niche expansion (Title et al., 2024). For instance, many snakes prey on species that are more active or breed during periods of high precipitation: amphibians require water for reproduction, while lizards feed predominantly on invertebrates that become more abundant during the rainy season (Oda, Bastos & Sá Lima, 2009; França & Braz, 2013; Alvarenga et al., 2017; Godinho, 2019). Indeed, our species lists reveal that most species in this study disproportionately prey upon amphibians and lizards (*e.g.*, *Erythrolamprus* and *Chironius* species). For species that fluctuate independently with no compensatory dynamics, one possible explanation is that the Cerrado-Amazonia transition is known to harbor a greater diversity of plants, including species from both forest and open habitats, creating a highly heterogeneous ecosystem (Lenza et al., 2024) that can facilitate the colonization of highly diverse functional groups of snakes. The species we recorded present complexity of behavioral traits, with cryptozoic, terricolous-cryptozoic, or cryptozoic-fossorial species comprising 5.5% of the total, followed by strictly arboreal, sub-arboreal-terricolous, and strictly fossorial (7.5%), arboreal/semi-arboreal (15%), aquatic (11.5%), and terricolous (34.5%). Some species are generalists (*e.g.*, *Drymarchon corais*, *Boa constrictor*), whereas others are more specialized (*e.g.*, *Micrurus frontalis*, *Tantilla melanocephala*). Thus, the apparent lack of intense competition for resources—possibly due to niche flexibility or resource partitioning—may lead to positive covariance and a lack of synchrony between species.

While our findings cannot directly confirm or refute the presence of competition within the system, the observed patterns suggest that other factors may play a larger role in shaping community structure. Snakes tend to have a broad dietary niche with generally non-overlapping diets, which may reduce potential competition, as indicated by the disproportionate consumption of vertebrate prey and a weak signal of trait divergence across varying ecological conditions (Burbrink & Myers, 2015; Title et al., 2024). This weak signal could reflect influences such as ecological specialization or historical constraints rather than direct competition for resources. Therefore, processes such as environmental filtering or niche differentiation may be more influential in structuring the community over time. Moreover, the community composition may rarely achieve long-term equilibrium due to drastic seasonal environmental changes, which can weaken or obscure the signal of competition.

It is essential to note that three species—*Erythrolamprus poecilogyrus*, *Pseudoboa nigra*, and *Dipsas mikanii*—were particularly abundant and dominated the community over time, with complex dynamics between seasons. The dry seasons of 2019 and 2020 exhibited significant community convergence, driven by the dominance of well-adapted species, including *P. nigra*, *E. poecilogyrus,* and *D. mikanii*. These species are well-adapted to anthropogenic pressures and are commonly found in disturbed areas (Nogueira et al., 2019). Land use in Nova Xavantina is intense, favoring species that tolerate disturbed habitats. Such dominance by a few species may reflect their ability to tolerate resource scarcity and thrive under the stressful environmental conditions typical of dry periods. During these seasons, other species with lower tolerance to drought or anthropogenic pressures may have declined or disappeared, contributing to high turnover rates dominated by species losses.

In contrast, the wet seasons of 2018–2019 and 2019–2020 were marked by community divergence, as increased rainfall and resource availability facilitated the appearance and persistence of a greater number of species, increasing interaction and reducing the relative dominance of the three resilient species. The MRS results further emphasize the dynamic interplay between species’ adaptations and seasonal environmental conditions. *Pseudoboa nigra* and *Erythrolamprus poecilogyrus* both showed reduced detections in the wet season of 2019, coinciding with a peak in turnover rates driven by high species appearance. These results suggest that increased biotic interactions during the wet season may disadvantage dominant species that thrive under harsher conditions. Similarly, *D. mikanii* increased in abundance during the 2020 dry season, potentially benefiting from reduced interaction.

## Conclusion

The ecotonal zone studied has proven to be a favorable region for both savanna and forest snake species, exhibiting a rich snake community. However, our temporal dynamics analysis suggests that continuous deforestation and habitat fragmentation in the region might influence stability over time due to the homogenization of the community. Notably, two species identified in this study are potential new species, but both were represented by only a single individual, highlighting the need for further investigation. The lack of recent encounters with these species raises concerns about local extinctions caused by ongoing environmental changes. Therefore, data comprising extended time series in the region and other Cerrado ecotonal areas are necessary. Such efforts are crucial for understanding and mitigating the impacts of habitat degradation on snake biodiversity, as well as for ensuring effective conservation strategies for these ecologically significant regions.

## Supplemental Information

10.7717/peerj.20025/supp-1 Supplemental Information 1 Rarefaction and sample coverage for dry seasons sampled (A) Rarefaction result for the dry season of 2018; (B) Sample coverage for the dry season of 2018; (C) Rarefaction result for the dry season of 2019; (D) Sample coverage for the dry season of 2019; (E) Rarefaction result for the dry season of 2020; (F) Sample coverage for the dry season of 2020.

10.7717/peerj.20025/supp-2 Supplemental Information 2 Rarefaction and sample coverage for wet seasons sampled (A) Rarefaction result for the wet season of 2018; (B) Sample coverage for the wet season of 2018; (C) Rarefaction result for the wet season of 2019; (D) Sample coverage for the wet season of 2019; (E) Rarefaction result for the wet season of 2020; (F) Sample coverage for the wet season of 2020.

10.7717/peerj.20025/supp-3 Supplemental Information 3 Data for rarify analysis The total abundance of each species used to calculate the sampling efforts.

10.7717/peerj.20025/supp-4 Supplemental Information 4 Data for temporal analyses The whole capture history of each species by their abundance and the seasons sampled during the study to perform the temporal dynamics analyses.

10.7717/peerj.20025/supp-5 Supplemental Information 5 Author checklist

10.7717/peerj.20025/supp-6 Supplemental Information 6 R code The code used for developing the analysis to investigate the temporal dynamics of the snake community.

## Additional Information and Declarations

### Data Availability

The following information was supplied regarding data availability: The raw measurements and code are available in the Supplemental Files.
