# Supplementary figures and images for: On the composition and temporal dynamics of a snake community at the Cerrado-Amazonia ecotone
Source: PeerJ. 2025 Sep 23;13:e20025. doi: 10.7717/peerj.20025 (PMC12466494; doi:10.7717/peerj.20025)

A

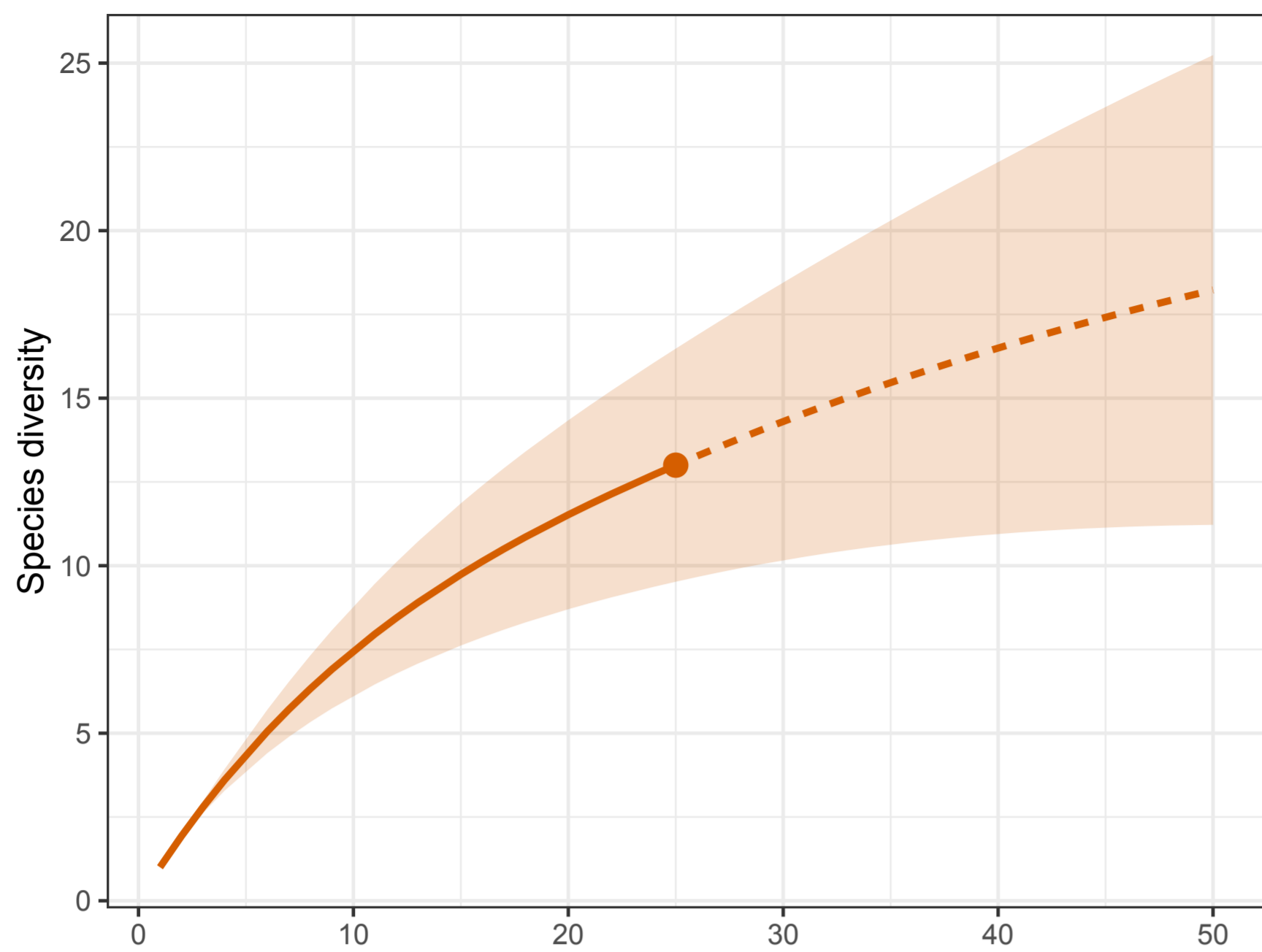

B

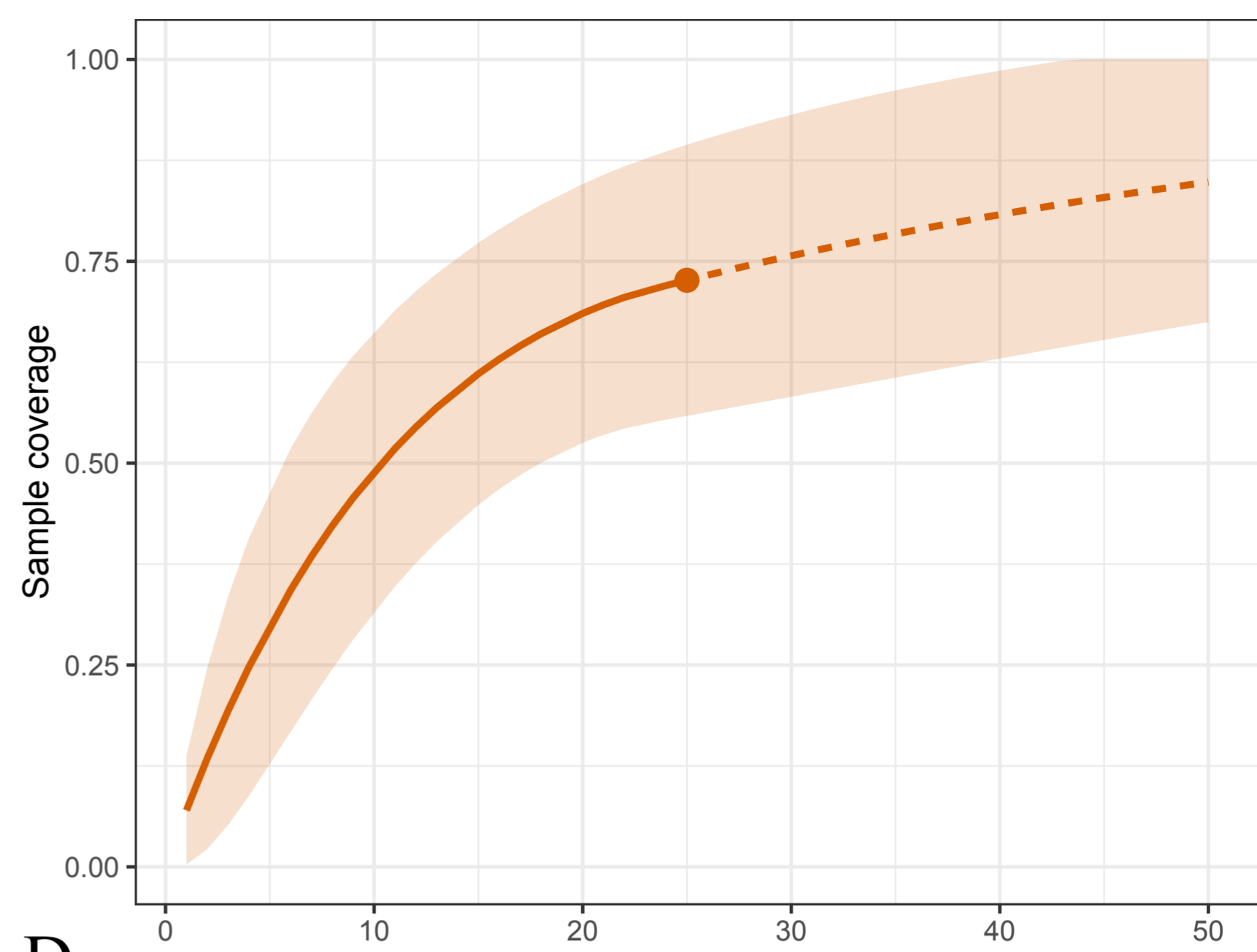

C

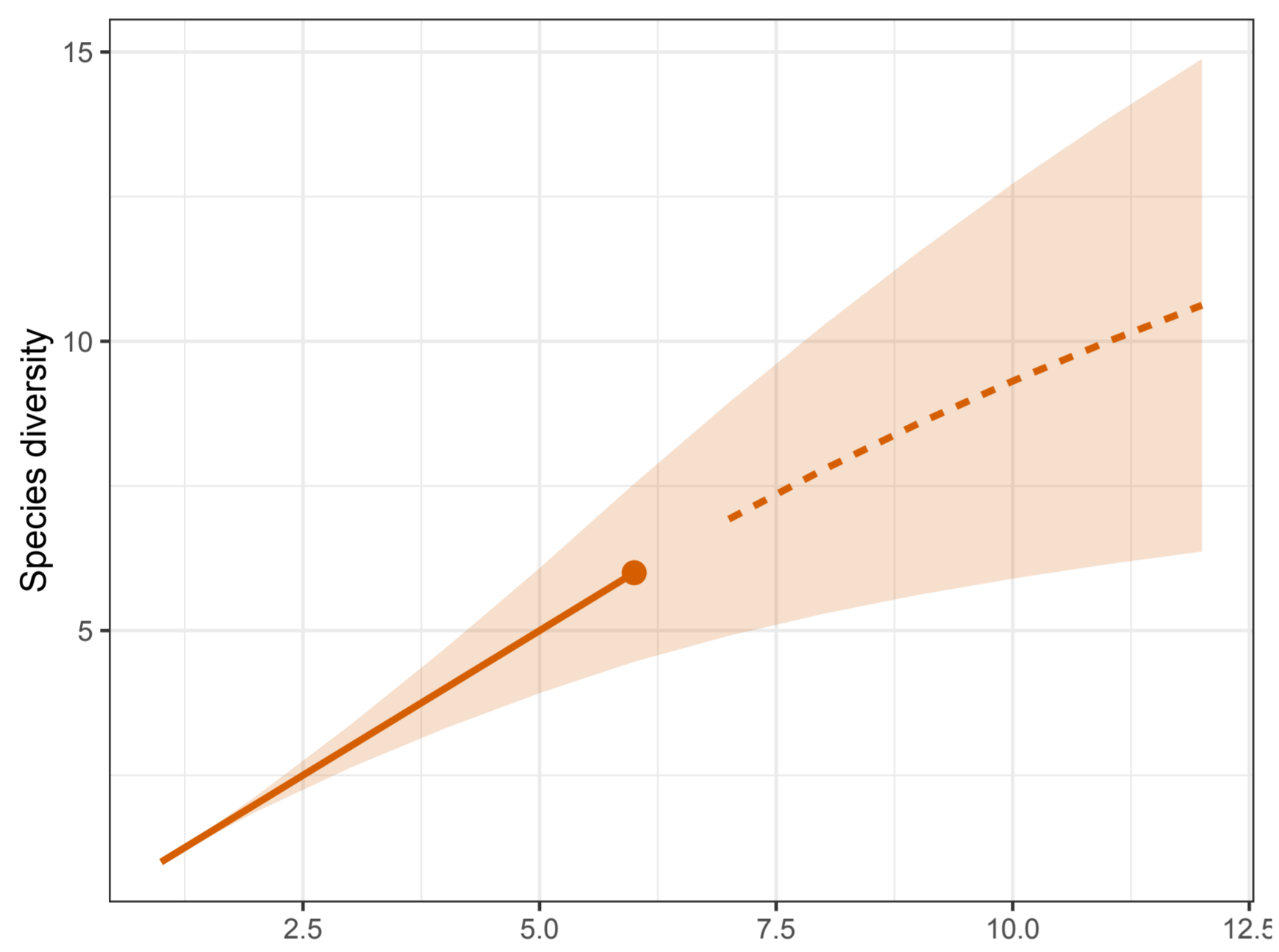

D

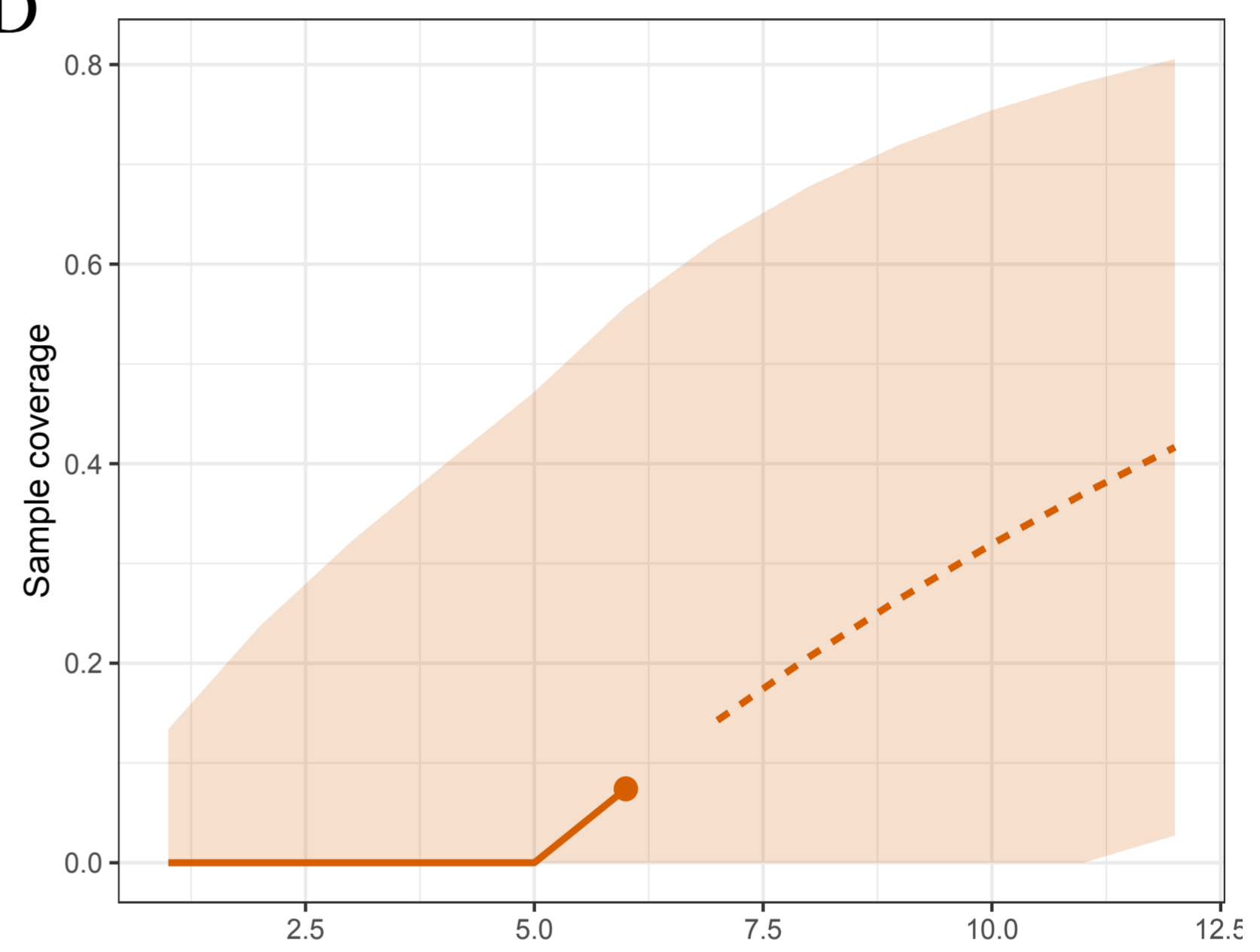

E

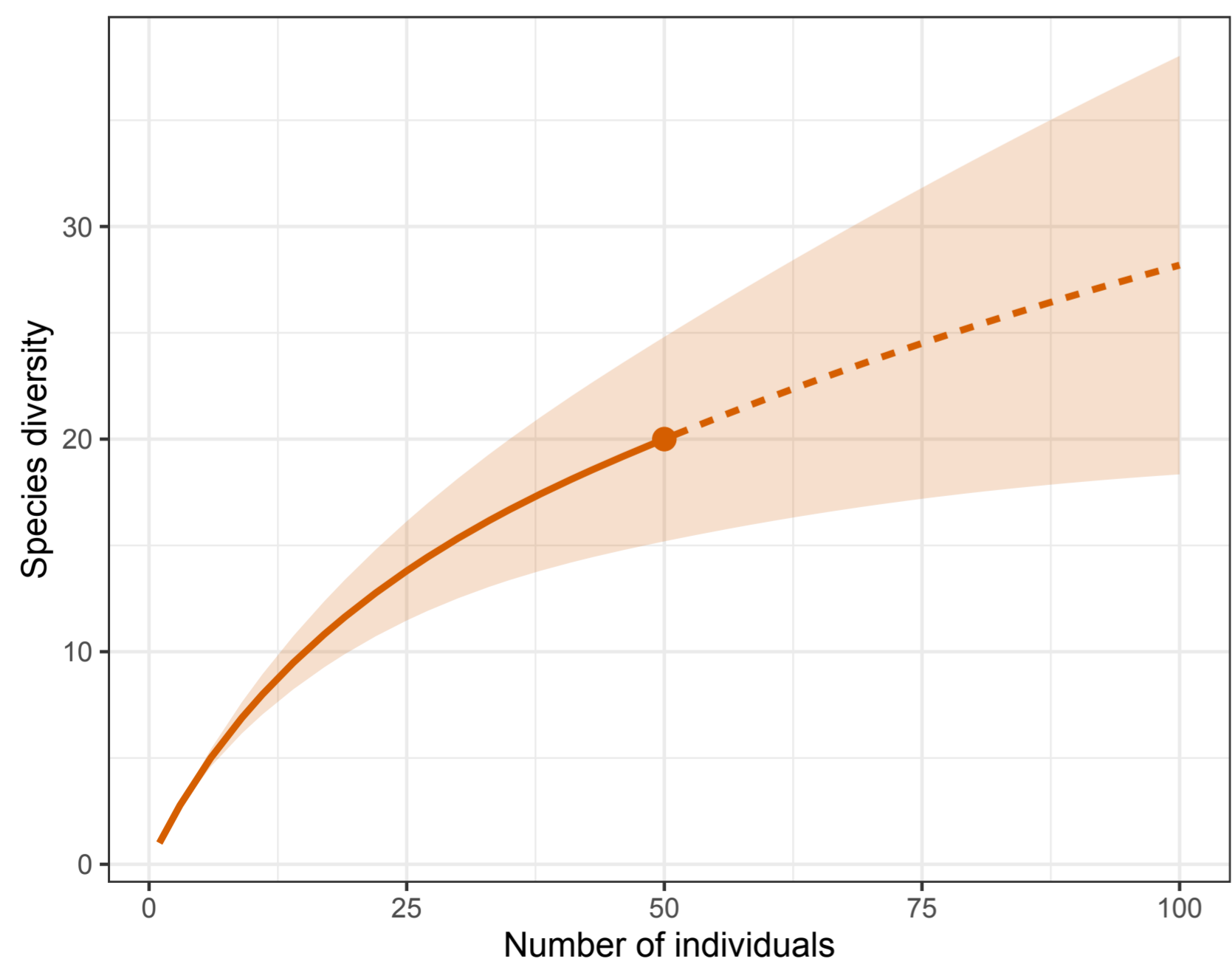

F

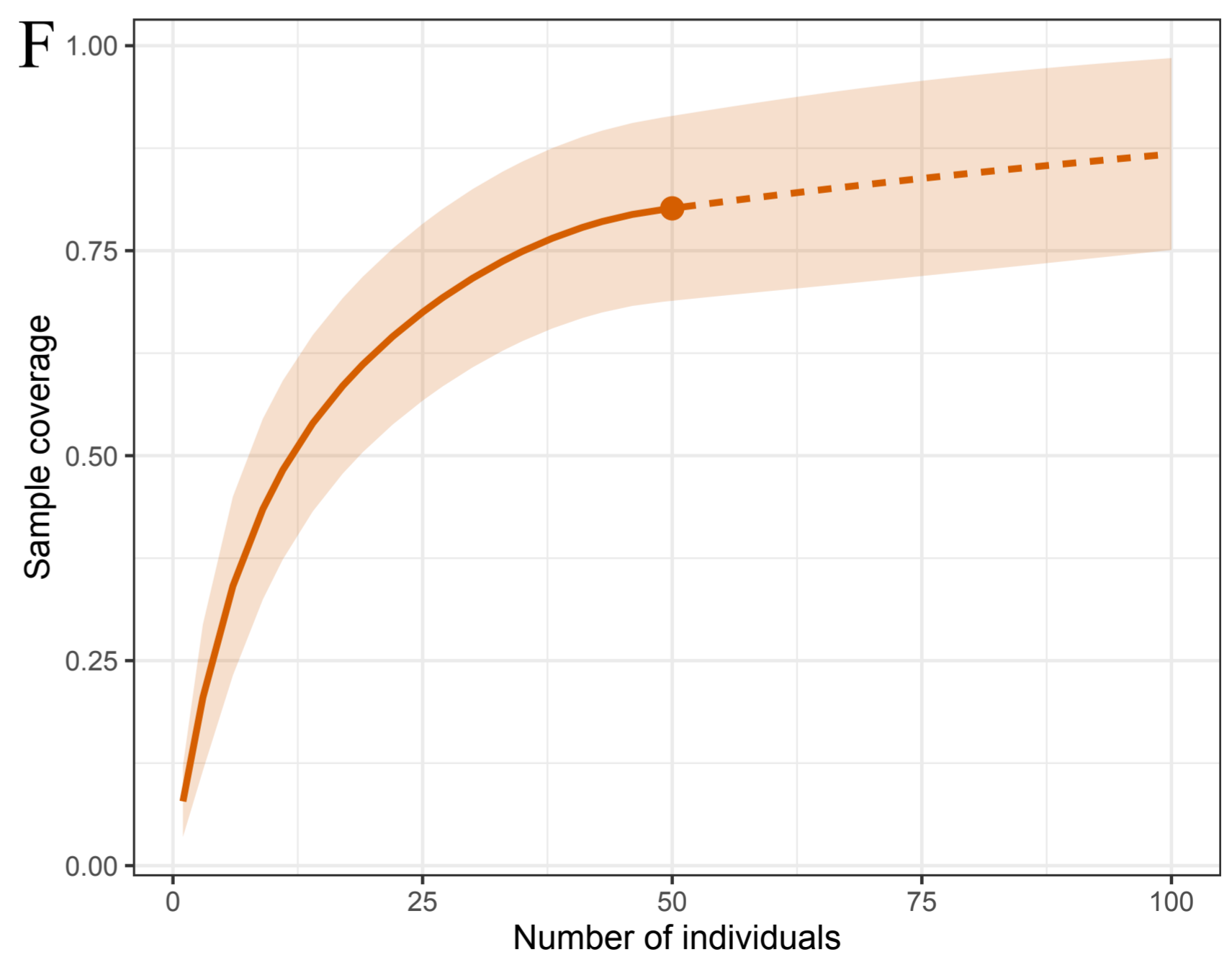

Supplement: Supplemental Information 1 — (A) Rarefaction result for the dry season of 2018; (B) Sample coverage for the dry season of 2018; (C) Rarefaction result for the dry season of 2019; (D) Sample coverage for the dry season of 2019; (E) Rarefaction result for the dry season of 2020; (F) Sample coverage for the dry season of 2020. [file peerj-13-20025-s001.pdf]

A

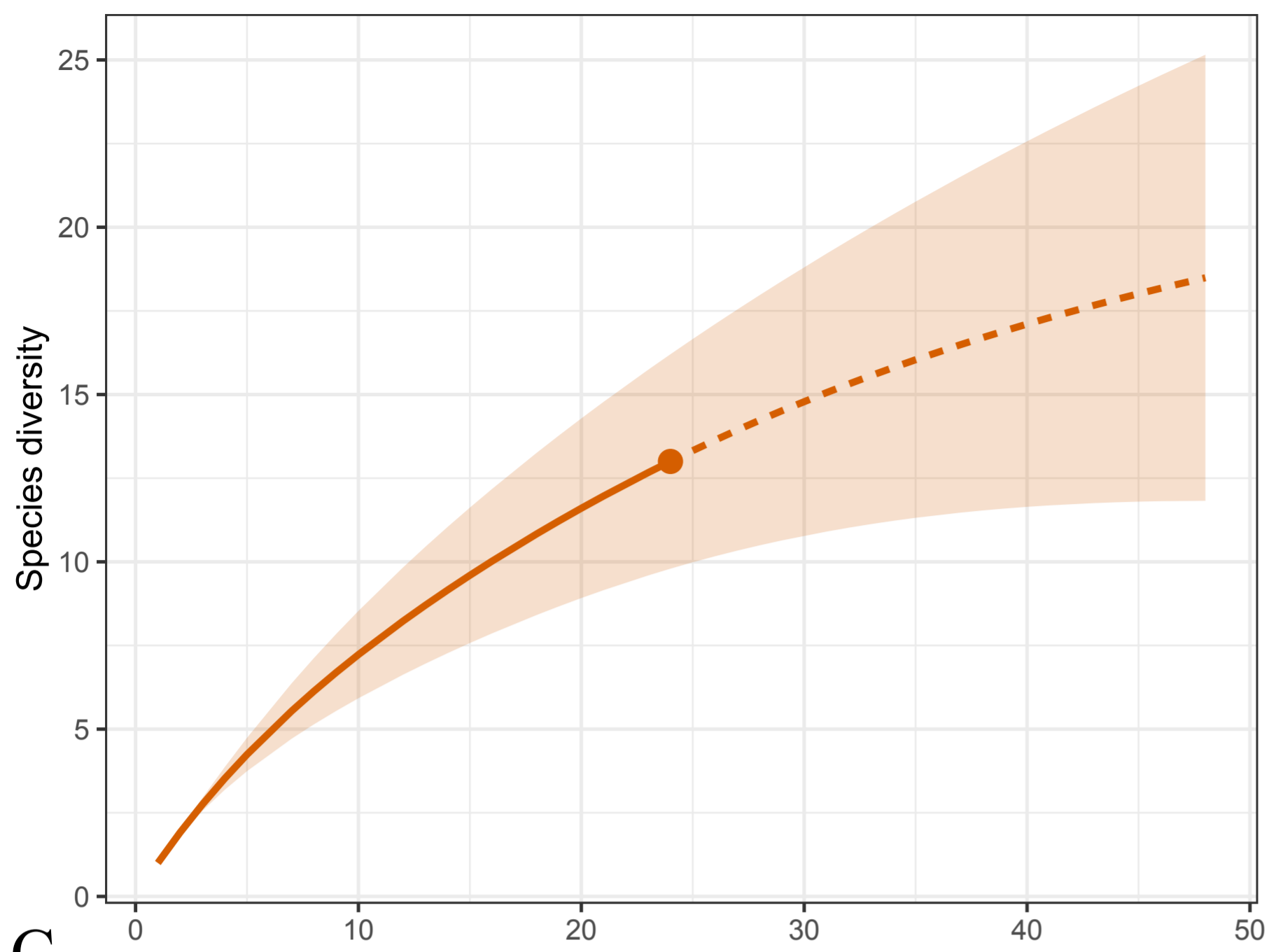

B

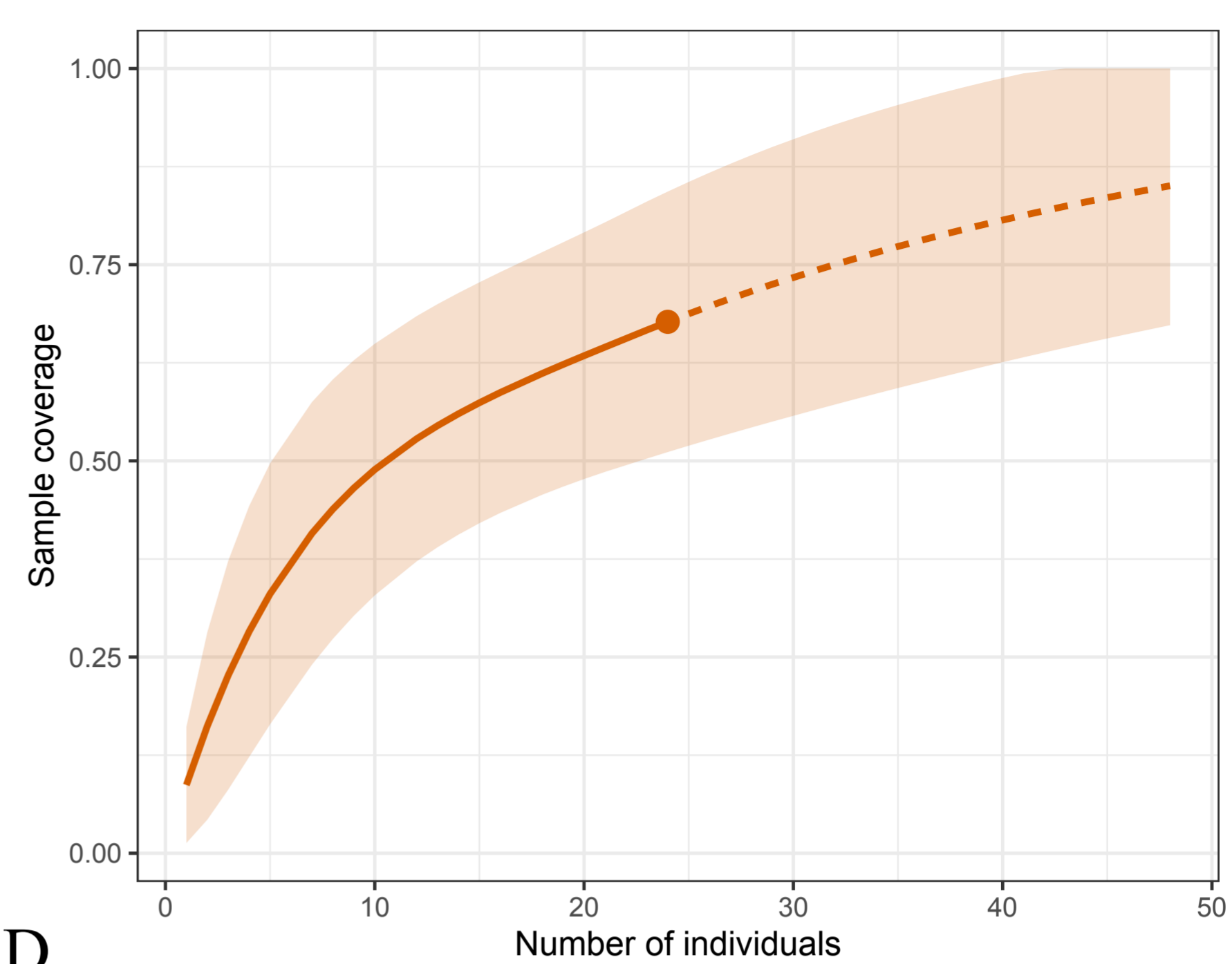

C

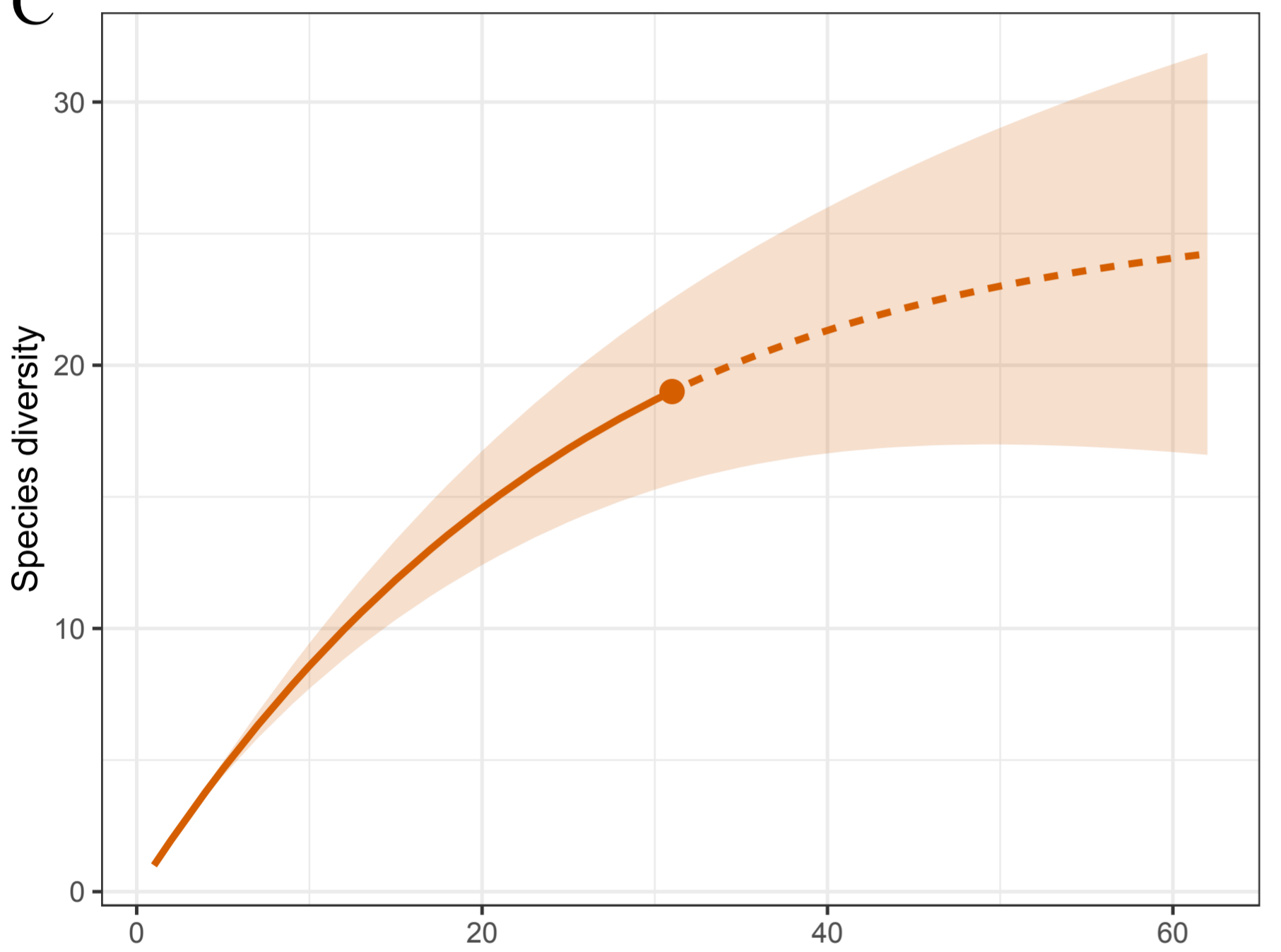

D

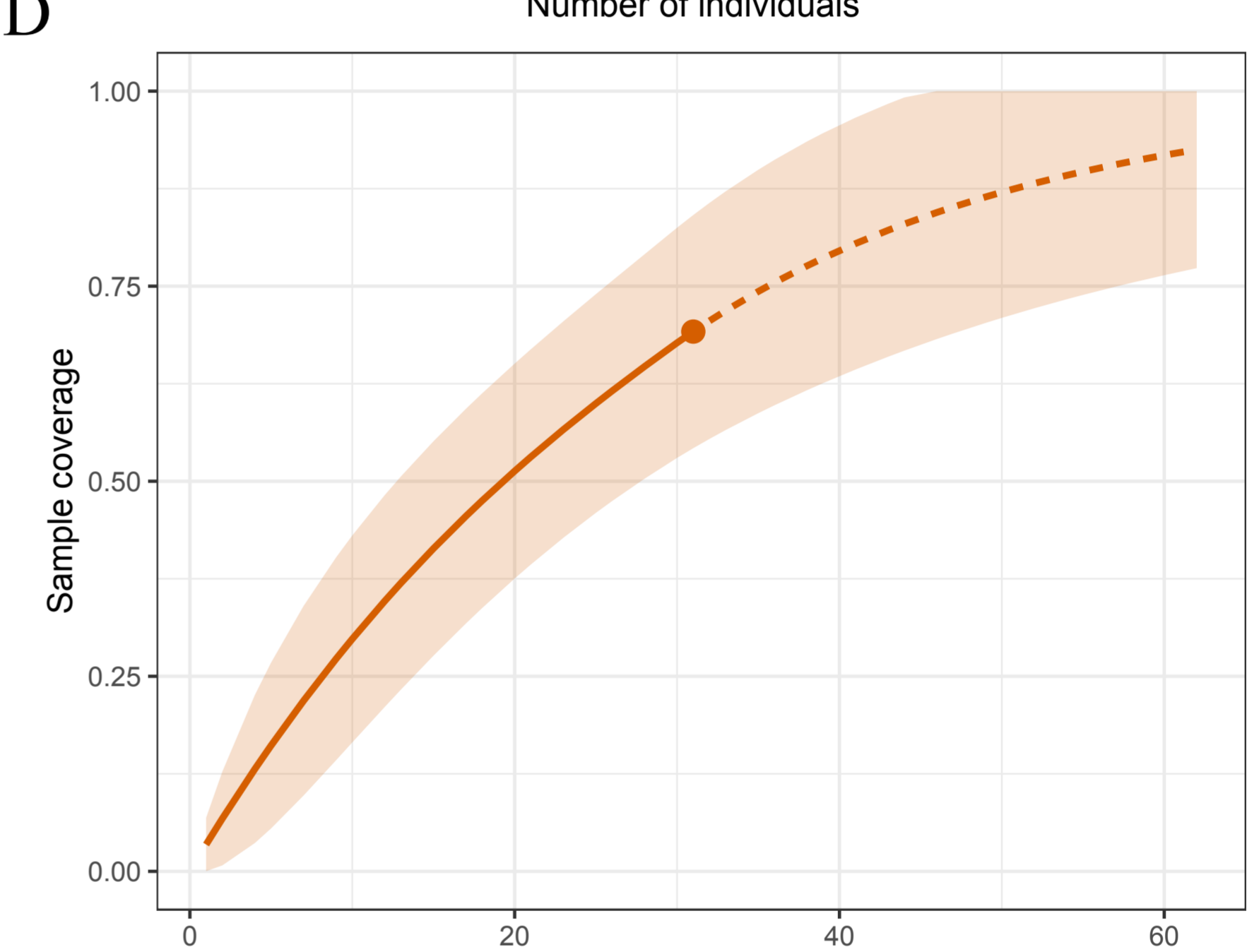

E

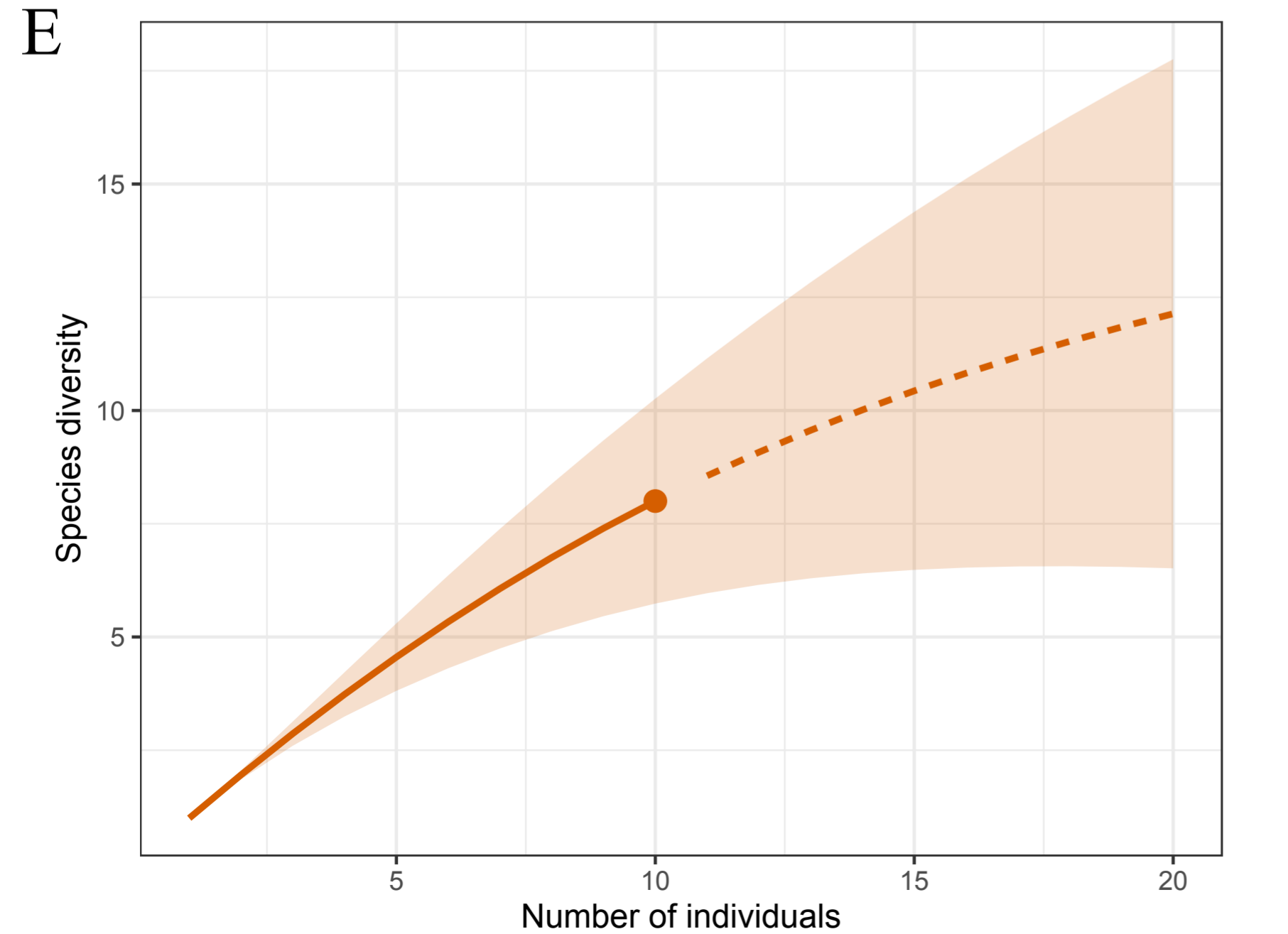

F

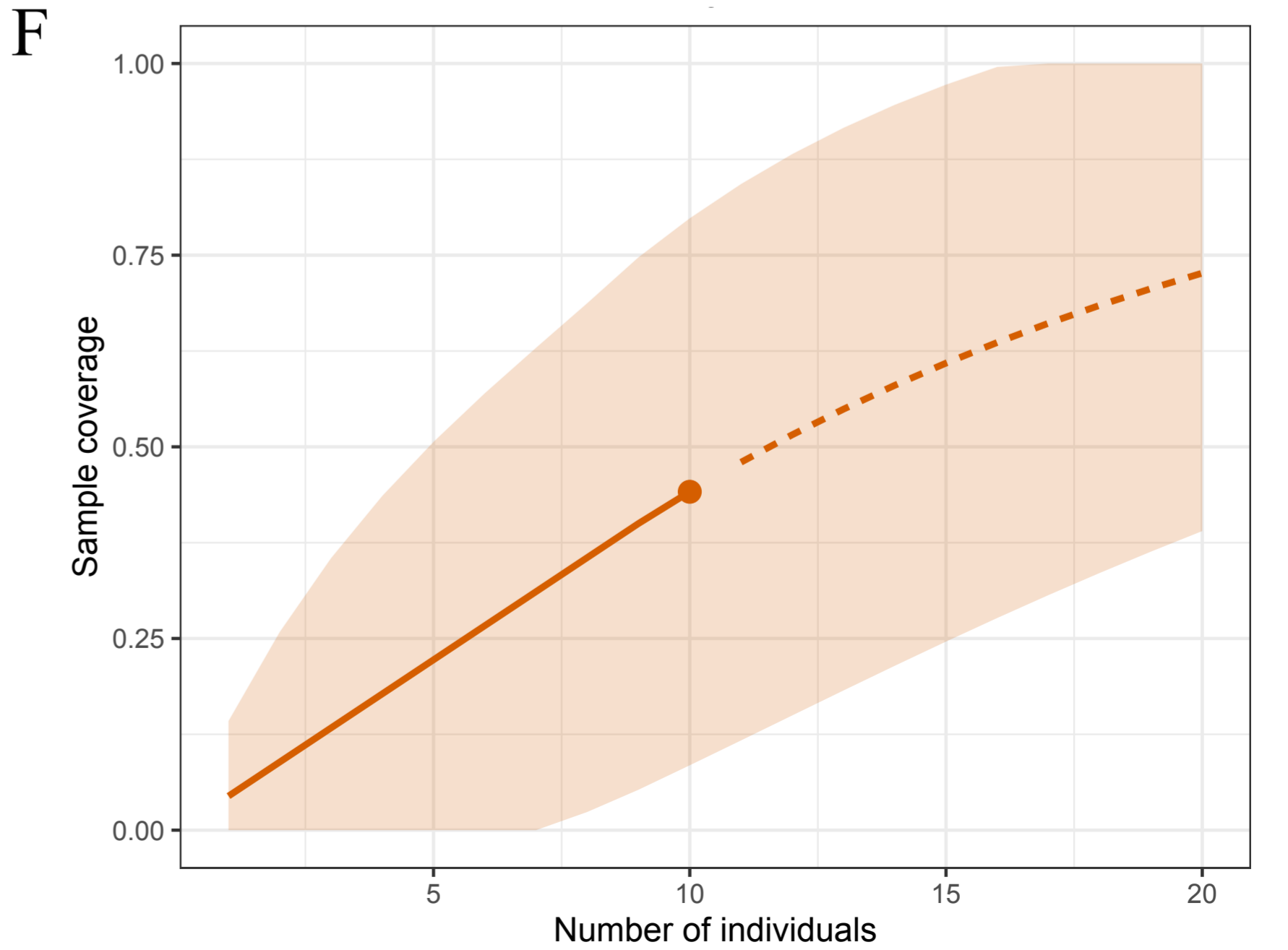

— Rarefaction    - · - Extrapolation    ● 0

— Rarefaction    - · - Extrapolation    ● 0

Supplement: Supplemental Information 2 — (A) Rarefaction result for the wet season of 2018; (B) Sample coverage for the wet season of 2018; (C) Rarefaction result for the wet season of 2019; (D) Sample coverage for the wet season of 2019; (E) Rarefaction result for the wet season of 2020; (F) Sample coverage for the wet season of 2020. [file peerj-13-20025-s002.pdf]
